# Untroubling abortion: A discourse analysis of women’s accounts

**DOI:** 10.1177/0959353517696515

**Published:** 2017-03-30

**Authors:** Siân M Beynon-Jones

**Affiliations:** University of York, UK

**Keywords:** abortion, women’s experiences, discourse analysis, subject positions, stigma

## Abstract

In this paper, I highlight key differences between a discourse analytic approach to women’s accounts of abortion and that taken by the growing body of research that seeks to explore and measure women’s experiences of abortion stigma. Drawing on critical analyses of the conceptualisation of stigma in other fields of healthcare, I suggest that research on abortion stigma often risks reifying it by failing to consider how identities are continually re-negotiated through language-use. In contrast, by attending to language as a form of social action, discursive psychology makes it possible to emphasise speakers’ capacity to construct “untroubled” (i.e. non-stigmatised) identities, while acknowledging that this process is constrained by the contexts in which talk takes place. My analysis applies these insights to interviews with women concerning their experiences of having an abortion in England. I highlight three forms of discursive work through which women navigate “trouble” in their accounts of abortion, and critically consider the resources available for meaning-making within this particular context of talk. In doing so, I aim to provoke reflection about the discursive frameworks through which women’s accounts of abortion are solicited and explored.

In recent years, stigma has become a central focus within research concerning women’s experiences of abortion (Hoggart, 2015; Purcell, 2015). Drawing on Goffman’s (1963) analysis of the social construction of stigma, Kumar, Hessini and Mitchell (2009) define abortion stigma “as a negative attribute ascribed to women who seek to terminate a pregnancy that marks them, internally or externally, as inferior to ideals of womanhood” (p. 628). In connecting abortion stigma to gender norms, they emphasise that understandings of abortion are locally specific and open to deconstruction. Their analysis considers how abortion stigma is perpetuated, one route being through “framing discourses.” However, as Purcell (2015) highlights, although available discourses have clear implications for women who end their pregnancies, few studies have analysed the language that women *themselves* use to represent their experiences.

In this paper, I begin to address this issue, drawing on insights from discursive psychology (for example, Edley, 2001; Potter & Wetherell, 1987) to explore how women in England talk about having had an abortion during research interviews. In contrast to psychological approaches that treat language as a means to access a speaker’s “internal” identity, discursive psychology explores how identities are *achieved* through speakers’ language-use (Edley, 2001). A key consequence of this conceptual move is that identities are not understood as static, but as variable and context-specific: They are articulated through socially *available* discourses or “interpretative repertoires.” Interpretative repertoires are routinised “ways of talking about objects and events” [which means] “when people talk (or think) about things they invariably do so in terms already provided for them by history” (Edley, 2001, p. 198). Interpretative repertoires “position” speakers in particular ways (i.e. they imply specific kinds of identity), but speakers also exercise agency by taking up or rejecting different “subject positions” through their discursive labour (Edley, 2001).

Some subject positions are harder to claim than others because “departures from ‘what everybody knows to be appropriate’ require explanation and create ‘trouble’ in […] interaction” (Wetherell & Edley, 1998, p. 161). By exploring various forms of “trouble” that speakers encounter, discursive psychological research highlights the social norms that render particular identities problematic *and* individuals’ capacity to resist stigmatisation and construct “untroubled” subject positions (Reynolds & Wetherell, 2003; Reynolds, Wetherell, & Taylor, 2007; Wetherell & Edley, 1998).

In the analysis that follows, I explore the discursive work through which women successfully navigate the “trouble” they encounter when describing themselves as someone who has had an abortion. In doing so, I critically explore the discursive resources available for meaning-making within a particular context of talk about abortion. I also contrast a discourse analytic approach to women’s accounts of abortion with the very different approach taken by the growing body of literature that explores women’s experiences of abortion stigma. By juxtaposing these approaches, I seek to provoke reflection about the implications of the discursive frameworks through which women’s accounts of abortion are solicited and explored.

## Accounts of stigma

Several studies have used interview and survey data to explore how stigma features in women’s experiences of abortion (Astbury-Ward, Parry, & Carnwell, 2012; Cockrill & Nack, 2013; Cockrill, Upadhyay, Turan, & Greene Foster, 2013; Hoggart, 2017; Major & Gramzow, 1999; Shellenberg et al., 2011; Shellenberg & Tsui, 2012). For example, Cockrill and Nack (2013) identify three “manifestations” of abortion stigma in women’s interview accounts of their experiences of abortion in the United States. “Internalised stigma results from a woman’s acceptance of negative cultural valuations of abortion” (Cockrill & Nack, 2013, p. 974). “Felt stigma” is a woman’s anticipation of “unsupportive reactions to disclosing an unplanned pregnancy, an abortion decision, or an abortion history” (Cockrill & Nack, 2013, p. 980). “Enacted stigma” is “a woman’s experiences of clear or subtle actions that reveal prejudice against those involved in abortion” (Cockrill & Nack, 2013, p. 974). Cockrill and Nack illustrate strategies women use to individually “manage” such stigma, for example, through non-disclosure, through constructing their reasons for abortion as “exceptional,” or through condemnation of anti-abortion perspectives. They argue such strategies often perpetuate abortion stigma by making abortion invisible, or by suggesting that it requires exceptional grounds. Drawing on these qualitative findings, Cockrill et al. (2013) develop a scale to quantitatively measure the extent of individual-level abortion stigma.

Such research provides a valuable means of representing the impact of the stigmatisation of abortion on women’s lives. For example, in a study conducted in England and Wales, Astbury-Ward et al. (2012) argue that “women’s perceptions of abortion as a deeply discreditable and personally stigmatising event” (p. 3144) prevent them from seeking crucial social support. However, it is important to reflect on the understandings of language, identity and stigma which this body of research works with and reproduces. Language is approached as a “transparent medium” (Wetherell, 2001, p. 16) through which aspects of identity, conceived of as internal to individuals (e.g. attitudes, experiences, values, and perceptions), can be accessed by researchers as opposed to an ongoing social practice through which identities are *constructed* (Potter & Wetherell, 1987). Relatedly, stigma is conceptualised as a “negative attribute” and treated “as though it were *a kind of thing* […] a relatively static characteristic or feature, albeit one that is at some level culturally constructed” (Parker & Aggleton, 2003, p. 14 – emphasis in original), which can be measured using interview and survey accounts.

Writing in relation to other forms of stigma, Parker and Aggleton (2003) and Farrugia (2009) draw attention to the consequences of these kinds of conceptual formulation. Treating stigma as a “kind of thing” (Parker & Aggleton, 2003, p. 14) that can be measured through individual accounts of experience risks obscuring the *social* relations that produce stigmatisation, and make available particular responses to it. It also positions those who are subjected to stigmatisation in a particular and limiting way, as individuals who *possess* and have to “manage” a socially “spoiled” identity (Farrugia, 2009; Parker & Aggleton, 2003). In contrast, post-structuralist accounts of subjectivity (for example, Butler, 1990; Foucault, 1978) offer a means to understand stigmatisation as reproducing social relations of power that depend on the differentiation of “normal” from “deviant” identities through discourse (Farrugia, 2009; Parker & Aggleton, 2003). Crucially, this process of identity construction is conceptualised as ongoing and re-negotiable, making it possible to consider the capacity of those who are subjected to processes of stigmatisation to construct positive identities (Farrugia, 2009; Parker & Aggleton, 2003). In this paper, I explore what it might mean to apply these insights to women’s interview accounts of abortion, drawing – as outlined above – on the particular approach to discourse analysis provided by discursive psychology.

## Abortion in public discourse in Great Britain

In England and Wales, abortion is a criminal offence under the Offences Against the Person Act 1861, unless two doctors agree that it is necessary on one of several grounds that concern risks to the pregnant woman’s (mental or physical) health, or the future health of her foetus. These grounds were introduced through the Abortion Act 1967, a piece of legislation that has enabled abortion to “become entrenched as a normal part of routine healthcare” (Sheldon, 2016, p. 344) in Great Britain.^1^

Although it has facilitated liberal abortion provision, the Abortion Act constructs abortion as a “deviant” practice which requires regulation and positions women as incapable of reproductive decision-making (Boyle, 1997; Fyfe, 1991; Jackson, 2001; Lee, 2003, 2004; Sheldon, 1997, 2016). Negative framings of abortion are also generated by an entrenched anti-abortion lobby that depicts foetuses as individual persons (Franklin, 1991) and women who have abortions as the unwitting victims of a procedure which inevitably leaves them physically and psychologically damaged (Amery, 2014; Hoggart, 2015; Hopkins, Reicher, & Saleem, 1996). These framings dominate the British print media, which constructs abortion as a moral “controversy” and portrays the transgression of the feminine norm of maternity as a risky decision, associated with regret and suffering (Purcell, Hilton, & McDaid, 2014).

## Women’s experiences with abortion in Great Britain

Qualitative research concerning women’s experiences with abortion in Great Britain has focussed primarily on processes of abortion decision-making and of using healthcare services. A consistent finding is that women reach decisions about their pregnancies based on the specific relational contexts of their own lives (Hoggart, 2012; Lattimer, 1998; Lee, 2004; Lee, Clements, Inghan, & Stone, 2004; Purcell, Cameron, et al., 2014). In contrast with the law’s medicalised construction of abortion decision-making, this typically takes place *before* women approach healthcare professionals for help with accessing the procedure (Allen, 1985; Brown, 2013; Kumar, Baraitser, Morton, & Massil, 2004; Lee, 2004). While women do not generally want input into their decision-making, their interactions with healthcare services are nonetheless central in their experiences of their decisions as either socially legitimate or as problematic (Allen, 1985; Astbury-Ward et al., 2012; Harden & Ogden, 1999; Kumar et al., 2004; Lee, 2004; Purcell, Cameron, et al., 2014). This paper expands the focus of this existing literature by using discourse analysis to explore how women talk about having decided to have an abortion, and the subject positions available to them.

## Methods

Ethical approval for this study was obtained from an NHS Research Ethics Committee and from the University of York’s Economics, Law, Management, Politics and Sociology Ethics Committee. Study information was made available to women attending abortion clinics and, when recruitment in this context proved difficult, I also placed advertisements in non-clinical settings such as newspapers and social media. Regardless of the site of initial contact, the recruitment process was effectively the same. In both cases, women were provided with information about the study that explained how to contact me if they were interested in taking part. Seventeen women were recruited through the clinic route, and 11 through the non-clinic route. A gift of £20 was offered to thank women for their time. All participants provided informed consent.

The majority of participants had, or were pursuing, an undergraduate degree or professional qualification (n = 21). Using British census categories, most of the participants identified as “White British/Other White background” (n = 25). Two participants identified as “Black/Black British–Caribbean,” and one identified as having a specific “Mixed background.” With the exception of one participant, who had an abortion while living overseas, their experiences of abortion all took place in England. At the time of interview, nine women had children and 19 did not. The length of time since abortion varied considerably, from approximately three weeks to 37 years (with a mode of three to four weeks since first experience of abortion). This meant participants had very different opportunities to formulate narratives concerning their experiences. However, length of time post-abortion did not seem to be associated with differences in women’s discursive work.

Women’s age at the time of their (first experience of) abortion ranged from approximately 14–36 years, and this variable did appear to shape participants’ positioning of themselves during interviews. Resonating with studies which highlight parental involvement in teenage women’s pregnancy outcomes (Hoggart, 2012; Lee, 2004), women’s descriptions of ending a pregnancy under the age of 18 (n = 3) were distinguished by depictions of abortion as a decision that had been made by others. These accounts were so different from the rest of the data corpus that they are not explored in the analysis that follows, which focuses on how women negotiated the meaning of having decided to end a pregnancy.

Interviews took place by phone (n = 12) or face-to-face (n = 16). In recognition of the researcher’s role in making particular subject positions available to participants (Taylor, 2001), I tried to produce a supportive research encounter that did not replicate the “troubling” of abortion. Recruitment materials highlighted the absence of women’s voices from discussion of abortion and described study participation as a way to address this problem by building research knowledge about “the issues important to women.” In interviews, I tried to avoid suggesting that abortion needed to be justified by asking women to tell me about their experiences rather than asking “why” they had decided on abortion. I began with a very open-ended question (Can you tell me a bit about what happened when you first thought you might be pregnant?) to give women the opportunity to shape the focus of the conversation. I also used a topic guide to explore some pre-defined issues with all participants if they did not arise spontaneously (for example, aspects of the abortion care pathway, or views about media coverage of abortion). Throughout the analysis that follows, I foreground my role in the production of data by indicating when a question directly preceded a participant’s stretch of talk and by reflecting on my framing of the research.

Interviews were recorded and professionally transcribed verbatim. Owing to a recording device failure, one interview could not be included in the data corpus, but nonetheless informed the analysis. All participants’ identities have been anonymised using an interview number. As I have described elsewhere (Beynon-Jones, 2015), the use of numbers rather than pseudonyms represents a (far from ideal) solution to concerns that some women expressed about the concealment of their identities.

My analysis of the data was supported by the qualitative data management software NVivo 10. I explored the different forms of interactional trouble which women encountered and coded the transcripts in terms of thematic patterns in the discursive work via which they navigated this. Below, I present three examples of this discursive work and explain how it enables women to take up, or reject, particular subject positions in specific moments of talk concerning abortion. I also highlight how the positions available to women are limited by available “interpretative repertoires” (routinised ways of speaking) about abortion.

As discourse analysis is itself a form of social action, its proponents do not typically seek to make truth claims. Nonetheless, there are criteria against which the legitimacy of a discourse analysis can be assessed. In analysing my data, I was particularly concerned with “participants’ orientation” (Potter & Wetherell, 1987, p. 170), i.e. whether the forms of discursive labour that I identified seemed significant for women themselves, and “fruitfulness” (Potter & Wetherell, 1987, p. 171), i.e. whether the discursive patterns identified provided new analytic insights.

## Analysis

### Asserting certainty

In this section, I illustrate a key form of discursive labour in which women engaged, namely, the strategies via which they positioned themselves as “certain” that their decisions to end their pregnancies were correct. The articulation of abortion as the only possible and legitimate outcome of a particular pregnancy was central to most women’s accounts. However, what was notable about these accounts was that assertions of “certainty” were often constructed through the refutation of alternative, “troubled,” subject positions:

**Extract 1:** It was totally fine. It was kind of, you know, because I was 100 per cent sure about my decision I was fine. I really didn’t think about it. There was no trauma about it. And then this year, no last year […] I found out I was pregnant again because I was a week late with my period and I was [*abroad*] and when I came back immediately I called. Did again a Google, called up, set up an appointment immediately and dealt with it a lot earlier this time […] So and, again, I currently – I’m turning [*age in mid-30s*] next month, I have no plans to have children and I’m in a place in my career where I’m just, it would be impossible. And I feel very confidently about my decision and I have no regrets and it’s been fine. (Interview 27. Note on transcription: […] denotes omitted text, [*italicised text in brackets is my annotation*], [non-italicised text in brackets represents unclear word(s)].)

**Extract 2:** So I went to the doctor’s the next day. And for me there was no doubt in my mind of what I was going to do. There was never any sort of question of, “Oh it’s a life, am I doing the right thing?” For me it was almost as if I was ill and I was going to the doctors to get better. It felt like that. It never felt like a big kind of dramatic soul-searching operation kind of thing. It just was kind of, “Oh gosh, I’ve got a problem and I need to get it solved.” I just – I think my doctor did as well. We both of us knew there was absolutely no way, the stage I was at in life, that I could possibly have gone through with it. I wanted to go to university. I was having a year out. As I explained I was - I had a lot going on at the time and for me there was just no doubt in my mind what I wanted to do. (Interview 20)

In both of these extracts, participants (implicitly or explicitly) navigate an anti-abortion repertoire of abortion as a dilemmatic decision linked to uncertainty and subsequent regret concerning the ending of foetal “life.” In the first, the word “fine” is used repeatedly to generate contrasts with subject positions of “trauma” and “regret.” In the second, the participant draws directly on the framing of abortion as a decision about “life,” but does not take up the subject position (of agonising decision-making about the morality of abortion, followed by subsequent regret) which is implied by this repertoire. In resisting this positioning, she repeatedly asserts her lack of “doubt,” drawing on the authority of medical opinion to reinforce the legitimacy of her decision.

Another way in which certainty is asserted in these accounts is through explanatory work around abortion decision-making. Such work would be anticipated by other studies that note the prevalence of justifications in women’s accounts of abortion (for example, Cockrill & Nack, 2013; Lattimer, 1998). However, an interesting feature of the explanations provided by participants in this study is that the desire not to have children, to pursue a career or to gain an education are depicted as legitimate goals which – far from requiring explanation or apology – are deployed as straightforward, untroubled, evidence of “certainty” about abortion. This resonates with Hoggart’s (2012) finding that, in spite of a UK policy discourse which constructs teenage pregnancy and abortion as problematic, teenage women are able to describe having an abortion as socially legitimate. In this study it was notable that participants across a wide range of ages at the time of abortion (18–36 years) constructed the pursuit of education, career, relationship or other life aspirations as valuable and, indeed, more legitimate alternatives to continuing with a particular pregnancy. Such accounts were provided by women with, and without, children.

Not all participants described abortion decision-making as non-dilemmatic. One participant, for example, spent nearly three hours discussing the difficult process of deciding to end her pregnancy following her partner’s decision not to support it. Nonetheless, while her account of the decision-making e*xperience* contrasts with those considered previously, she likewise engages in discursive labour to construct a subject position of retrospective “certainty”:

**Extract 3:** I don’t want to be one of these statistics that’s *another* young mum that’s single and on her own and has got all this baggage. And I want to be able to go back into education and do things and travel the world and go on holidays and things like that and not - Not that babies are a horrible thing, because they’re not, they’re the most wonderful gift in the world but it’s *when* you get them. […] I thought, “Oh my God I am the worst person in the world. I am going to be so ridiculously punished in some other life for this.” But at the end of the day, what’s worse? Is it worse to nip it in the bud, if you like, before it’s all come to fruition or to wait and have everything fall down around you, my relationship and everything, be on my own and not be able to cope and then get postnatal depression or something further along the line or resent that child or anything like that. And I think ultimately that’s worse than doing what I’ve done. So really, as much as I do feel sort of bad about it to a degree, I also feel that when the time comes it will be right and it will all be what it’s supposed to be.” (Interview 19)

This account provides a compelling illustration of the context-specific ways in which subject positions are achieved through talk, and the difficulties of treating language as a “transparent medium” (Wetherell, 2001, p. 16) through which to access an identity conceptualised as internal to individuals (such as “internalised stigma”). The participant’s suggestion that she thought she was “the worst person in the world” reads very differently when embedded in relation to the rest of her account. She invokes, and then resists, this stigmatised positioning to present herself as someone who has engaged morally with a difficult dilemma in order to reach the correct decision. Her assertion that she has made a legitimate choice is underscored through her juxtaposition of the catastrophic implications of continuing with her pregnancy vs. waiting for the “right time.” Similar depictions of the importance of “responsible” maternity are also highlighted by Hoggart (2017), who employs a very different analytic framework through which to conceptualise their significance in women’s accounts of abortion (see also Lattimer, 1998).

Women who did not have children, such as the participant speaking in the previous extract, typically (although not exclusively – see Extract 1) emphasised their plans to pursue maternity *in the future*. In other words, it often seemed to be the *temporariness* of their rejections of maternity that enabled women to position themselves as “certain” about abortion:

**Extract 4:** I don’t like regret it at all because obviously – if I’d had been with someone and lived with them [*The participant had previously described difficulties in her relationship with her partner which made maternity* “*impossible*”]. That’s what I think, I think if it affects me when I obviously do want to have children I’ll be absolutely devastated with the choice I made but. (Interview 21)

In this account, “regret” is temporally re-framed. Rather than applying to the decision to end a pregnancy (which is asserted as correct), it is constructed as a potential emotional state connected to the loss of future fertility in alternative circumstances. Such constructions draw on an enduring and incorrect (Royal College of Obstetricians and Gynaecologists (RCOG), 2011, p. 43) anti-abortion claim, namely, that abortion damages women’s fertility. Concern about future infertility was also central to the account of one of only two participants in the study who did not take up a subject position of “certainty,” instead framing abortion as a choice that she would not have made if given the opportunity to revisit it. As Hoggart (2012) notes, it is important that feminist discourse contains space to acknowledge regret about abortion. However, as all the extracts in this section illustrate, it is also vital to interrogate the discourses available to women in their attempts to negotiate the meaning of having ended a pregnancy (Hoggart, 2012, 2015; Kimport, 2012).

### Emphasising individual agency

Another key form of discursive work via which women untroubled the meaning of having decided to have an abortion was to critique others’ negative judgements. In analysing women’s strategies of stigma management in relation to abortion, Cockrill and Nack (2013) argue that:

Through *condemning the condemners*, a woman can assign the greater sin to those who have judged abortion to be wrong and who work to limit women’s access to abortion. This neutralizes the act of abortion by socially constructing the anti-abortion value system as more unjust and immoral than having an abortion. (p. 985–emphasis in original)

Adopting a discourse analytic perspective, I approach women’s accounts of condemnation slightly differently. Rather than assuming that they are used to “neutralise” an automatically negative identity, I explore them as illustrative of the context-specific repertoires available for identity construction. On what basis is it possible for women to critique others’ problematisations of abortion and what are the implications of this for the subject positions available to them?

In this study, a cross-cutting feature of such accounts was participants’ deployments of a repertoire of “liberalism”:

**Extract 5:** … [*a friend*] told a lot of people so now I’m getting a lot of stick […] for it. That’s probably the only reason why I said I’m definitely doing this interview now is just because I’ve had so much stick for my decisions and it’s got to a point now where I’m getting so infuriated with how people react to abortion. It’s just like, it was my personal choice. It’s not anything that you had to do. I didn’t force you to have an abortion of your child or anything like that so there’s no reason I should get people’s hate. (Interview 10)

**Extract 6:** My [*relative*] isn’t supportive of it [*abortion*] but at least he turned round and said, “If it’s the right decision for you then I will support you.” […] He’s done it the way that I’d want. He’s not said that he’s supportive of my decision but he’s supportive of me. Whereas she’s [*another relative*] done it in the way that, “No, this is wrong.” […] I will never speak to her again for what she said to me. (Interview 3)

In these extracts, women position themselves as making a personal choice/decision, which does not impact on the agency of other people. Conversely, those who express views that abortion is morally wrong to women who have ended their pregnancies are positioned as intruding harmfully into the individual experiences of others.

Women developed related critiques concerning their experiences of navigating anti-abortion protests outside of clinics:

**Extract 7:** Siân: And in terms of this research study was there a particular reason that you wanted to take part or not take part or?

Participant: I just kind of like doing it I guess. I think it helps. And it’s like really annoying when those people stand outside the clinic all the time. At the [*clinic*] there was the people that are really against abortion. And I just don’t think – like obviously they’re Christian or religious - but I don’t think they really have the right to be there because obviously at the end of the day it’s your choice. I think that kind of made me do it because I was like you don’t really understand the reasons why people do it. It’s not like I got pregnant and then just decided, “Oh yeah, I’ll just have an abortion for fun sort of thing.” That kind of annoyed me which is why I also think I took part because I don’t think that you should give people who have terminations a bad name. I don’t think that’s really fair because you don’t understand other people’s circumstances. […] I think the worst thing is that obviously it’s going to be a hard decision for some people anyway. I just don’t think – like there should be, I don’t know, some rule against it and they shouldn’t – like obviously I don’t mind if they want to express their views but not outside the place where it’s going to happen. And especially if you’ve got the pictures like the [*inaudible*] weeks and everything, that doesn’t help either. They’re there constantly throughout the day and then they were there the next day as well and I was like I just don’t want to deal with this really. (Interview 17)

In this extract, the participant engages in complex discursive work to construct the protestors’ presence outside of abortion clinics as illegitimate. In order to reconcile a liberal repertoire concerning the importance of understanding and accepting others’ personal positions, with the threat that the protestors pose to her own reproductive choice, she argues that they should be able to “express their views,” but that it is not appropriate for them to do so outside abortion clinics. This echoes Extract 6, in which the participant differentiates between the behaviour of people who oppose abortion, and those who *express* these views to women who have decided to end their pregnancies.

Critiquing particular expressions of opposition to abortion as oppressive encroachments into private choices and experiences enabled participants to depict their individual agency as morally important. It also allowed them to construct the problems they encountered as resolvable and to position themselves as potential agents in the process of addressing “ignorant” or “abusive” behaviour, by correcting misrepresentations of abortion. This positioning was, arguably, facilitated by the study’s explicit framing as a means of “giving voice” to women through building research knowledge about marginalised perspectives.

Nonetheless, liberalism also “dissolves entire areas of socio-political conflict into interpersonal problems” (Kitzinger, 1987, p. 196). Constructions of stigmatisation as the product of individually problematic behaviours (e.g. ignorance or abusiveness) render invisible – and make it harder to confront – the gendered social relations which construct abortion as a stigmatised course of action (this argument is outlined by Parker & Aggleton (2003) in the context of HIV/AIDS). Although the promotion of tolerance through education is widely advocated as a solution to multiple forms of stigmatisation, “tolerance and intolerance are […] very much the same thing – neither position requires those in power to give up power, rather these concepts reinforce power differentials by denoting who does and does not have the power to be tolerant” (Clarke, 2005, p. 4). Notably, in the extracts considered above, women position themselves as *dependent upon* others’ understanding and tolerance. However, it is important to note that a minority of participants also drew on an alternative repertoire to critique opposition to abortion. This located their experiences very differently, namely, as the result of gender inequalities:

**Extract 8:** I think women are demonised and it’s never anything to do with a man. As if like we just got pregnant by ourselves yeah, or immaculate conception. (Interview 12)

**Extract 9:** I think it’s a feminist issue really. It’s like I’m sure if men were having abortions and stuff it wouldn’t be such a taboo. (Interview 15)

### Explaining silence

While women regularly took up the position offered by the study’s framing, of voicing a missing perspective that should be central to discussion of abortion, many (although not all) constructed speaking *as* a “woman who has had an abortion” as difficult. As outlined in the introduction, existing research concerning abortion stigma has treated women’s accounts of silence as evidence of their attempts to manage a stigmatised identity, highlighting the harmful consequences of this strategy for women’s well-being. Below I suggest that, rather than simply using accounts of silence as evidence of stigma management, it might also be useful to explore women’s talk *about* silence. What discursive work do women engage in when they account for abortion non-disclosure? What subject positions does talk about silence make (im)possible? In adopting this approach, I draw on the work of Wigginton and Lafrance (2016), who demonstrate how available discourses limit the possibilities of speaking as a pregnant smoker, constituting this as a subject position which is – necessarily – “untellable” (p. 33). Similarly, I suggest a discourse analytic approach to accounts of abortion non-disclosure illustrates (and makes it possible to critique) social norms about identifying as a woman who has had an abortion.

Central to the way in which women described talking about having an abortion was the notion that there is a taken-for-granted etiquette concerning the circumstances in which this is “appropriate,” or “reasonable”:

**Extract 10:** Siân: And is it something that you sort of talk about with other people, your experiences of it or?

Participant: Yeah, I’ve talked about it with probably three of my closest friends but not - maybe four even – but not, not flippantly. But I have shared my experiences with friends, yeah.

Siân: Yeah, ok. So when you say not flippantly do you mean sort of it’s something that you’d have a more of an in-depth conversation about as opposed to something you would mention in passing kind of thing?

Participant: Yeah. No, “Oh yeah, I had one of those,” [*laughs*], “Oh I had two of them in fact!” [*parody*] More like I guess a couple of them – how did I talk about it actually? I think because I’m at an age where we talk about children and maybe in conversations I guess it came up where like, “Yes, I’ve been pregnant and I’ve had an abortion.” (Interview 27)

In this account, openness about abortion is positioned as entirely possible within the context of close friendships, but it is not something that can reasonably be mentioned in casual interaction. Indeed, my suggestion that abortion *could* be mentioned in this way is so “troubled” that it is constructed and dismissed as a joke.

Rules surrounding speech about abortion re-occurred in many of the interviews:

**Extract 11:** I wouldn’t talk about it at a dinner party but if I’m with some close girlfriends and it comes up then I’ll. Because it’s not something I’m embarrassed about, I think people understand the situation, but you obviously gauge your crowd and you know the reactions that you would want to, you know. (Interview 23)

**Extract 12:** Interviewer: […] is it something you talk to other people about or?

Participant: I think I told maybe sort of friends-wise only maybe one or two people. And if it comes up in conversation normally I just kind of say that we lost the baby. But then that kind of - I feel a bit ashamed to lie about it but I’m not lying it’s just, it’s such a big conversation to have and to try and explain that I just tend to say that we just lost the baby. But I think my gut instinct is that I would just say but it’s just that it’s such a big conversation. And then like we said, it’s the timing. If your friend is pregnant it’s not a conversation that you’re going to enter into. (Interview 5)

In these extracts (drawn from interviews in which the ending of a pregnancy held very different kinds of emotional meaning for participants), women’s positioning of themselves as “open” has to be carefully navigated in relation to admissions of non-disclosure in which they risk positioning themselves as “ashamed” or “embarrassed.” This trouble is resolved, I would argue, through the emphasis which women place on the “obviousness” of non-disclosure. Talking about abortion in particular contexts is described as an interactional impossibility, rather than a personal choice. Disclosing an abortion is not suitable at “dinner parties,” it cannot be discussed “flippantly,” it is a “big conversation” and there are clear circumstances in which this “is not a conversation you’re going to enter into.” However, while these constructions of “obvious non-disclosure” enable women to manage the rhetorical difficulties involved in accounting for silence, they also depict talking about having an abortion as *intrinsically* socially problematic.

## Conclusion

Drawing on insights provided by critical approaches to the conceptualisation of stigma in other fields of healthcare (Farrugia, 2009; Parker & Aggleton, 2003), this paper has highlighted the significance of the conceptual frameworks through which women’s accounts of abortion are explored. Treating such accounts as a “transparent medium” (Wetherell, 2001, p. 16) through which to access the “reality” of individual experiences of abortion stigma, I have suggested, risks reifying stigma as an a-social “kind of thing” (Parker & Aggleton, 2003, p. 14) and positioning women who have abortions as possessors of a “spoiled” identity. In contrast, discursive psychology treats identity construction as an unfolding social process, through which speakers position and re-position themselves in relation to particular contexts of talk.

In applying this approach to the analysis of women’s interview accounts of abortion in England, I have highlighted how the social contexts of talk about abortion shape women’s navigations of untroubled (i.e. non-stigmatised) identities. I have explored three examples of the discursive work in which women engage when talking about having decided to have an abortion. A central finding is the discursive labour in which women have to engage in order to negotiate an anti-abortion repertoire of (inevitable) regret and position themselves as “certain” about their decisions to end their pregnancies. Participants’ accounts illustrate that women *do* routinely articulate certainty about their decisions and that the desire to (albeit, often temporarily) pursue goals other than motherhood is often offered as a “common-sense” rationale for such certainty. Simultaneously, women’s accounts suggest that articulating certainty about abortion is *difficult* and requires the navigation of competing constructions of abortion.

In a country in which abortion is not a legal “choice” for women, it is striking that another key form of discursive work in which participants engaged was to emphasise the importance of non-interference with personal reproductive choices. Drawing on a repertoire of “liberalism,” women constructed abortion as a private decision and positioned others’ problematisations of abortion as individual failures of tolerance. While women’s capacity to prioritise their individual agency and autonomy is significant, I have suggested that it is important to reflect critically on the liberal repertoire through which this positioning is achieved. Constituting stigmatisation as “what some individuals do to other individuals” (Parker & Aggleton, 2003, p. 16) obscures, and makes it difficult to contest, the *social* relations through which particular subject positions become stigmatised.

I have argued that key insights into the social possibilities of meaning-making about abortion are also generated by attending to women’s talk about *not* talking about abortion. Most participants described not disclosing their abortion(s) in particular discursive contexts as an accepted, reasonable, taken-for-granted, social practice. De-constructing and challenging assumptions of “reasonableness” in relation to abortion non-disclosure (for example, that abortion cannot be easily mentioned in conversation, and that it is reasonable for women to take responsibility for censoring their speech in order to manage others’ reactions to their experiences) represents a potentially useful strategy for future feminist advocacy.

In comparison to conventional accounts of abortion stigma, addressing language as a form of social action both offers alternative insights into women’s accounts of abortion and underscores the significance of the discursive contexts in which these accounts are solicited. This study positioned “women’s voices” as marginalised but essential to understanding abortion, a framing which appeared to make particular positions available to participants. Women routinely asserted the legitimacy of abortion and highlighted the importance of their experiential knowledge. In terms of feminist research and abortion advocacy this illustrates the importance of attending not only to the production of spaces in which women can “voice experiences” but also the specific ways in which women are positioned through the framing of these spaces. What kinds of accounts of abortion do they make possible?

It is important to also highlight some of the silences produced through my discourse analysis of women’s stories. The relative demographic homogeneity of the sample (primarily White and/or highly educated) may have concealed social divisions in the discursive resources available for speaking about having an abortion in England. A focus on patterns that cut across women’s accounts also makes it easy to lose sight of other key differences, namely, participants’ very different emotional experiences of ending their pregnancies. Relatedly, while it offers important insights into the re-negotiable basis of identity, the analytic approach adopted in this paper arguably fails to consider what it is like to *live through* particular discursive positionings. It is because of my concerns about its inadequacy as a mode of engaging with the “painful lived experience of being stigmatised” (Farrugia, 2009, p. 1025) that I have not suggested discourse analysis should replace other approaches to the exploration of women’s accounts of abortion. Rather, because “writings, actions, practices and research on abortion are already carriers of political undertones” (Macleod, 2008, p. 67), I have argued that the frameworks through which we solicit and represent women’s accounts require greater reflexive interrogation.

## Note

1 The Abortion Act applies in Scotland, England and Wales (Great Britain) but not in Northern Ireland. Abortion in Northern Ireland is criminalised in most circumstances, leaving most women without access to the procedure in this country (Sheldon, 2016).
